# Angio-Based Fractional Flow Reserve, Functional Pattern of Coronary Artery Disease, and Prediction of Percutaneous Coronary Intervention Result: a Proof-of-Concept Study

**DOI:** 10.1007/s10557-021-07162-6

**Published:** 2021-04-08

**Authors:** Simone Biscaglia, Barry F. Uretsky, Matteo Tebaldi, Andrea Erriquez, Salvatore Brugaletta, Enrico Cerrato, Giorgio Quadri, Giosafat Spitaleri, Iginio Colaiori, Domenico Di Girolamo, Alessandra Scoccia, Ottavio Zucchetti, Emanuele D’Aniello, Marco Manfrini, Rita Pavasini, Emanuele Barbato, Gianluca Campo

**Affiliations:** 1 Cardiovascular Institute, Azienda Ospedaliero-Universitaria di Ferrara, Via Aldo Moro 8, 44124 Cona, FE Italy; 2 Central Arkansas VA Health System, Little Rock, AR USA; 3 University Hospital Clínic, Cardiovascular Clinic Institute, Institut d’Investigacions Biomèdiques August Pi i Sunyer (IDIBAPS), Barcelona, Spain; 4 San Luigi Gonzaga University Hospital, Orbassano and Infermi Hospital, Rivoli, Turin, Italy; 5 Interventional Cardiology Unit, S. Maria Nuova Hospital, Reggio Emilia, Italy; 6 Casa di Cura San Michele, Maddaloni, CS Italy; 7 GVM Care & Research, Maria Cecilia Hospital, Cotignola, RA Italy; 8 Department of Advanced Biomedical Sciences, Federico II University, Naples, Italy; 9 Cardiovascular Research Center, OLV Hospital, Aalst, Belgium

**Keywords:** Quantitative flow ratio, Angio-based fractional flow reserve, Percutaneous coronary intervention, Functional pattern of coronary artery disease, Pressure pullback gradient

## Abstract

**Purpose:**

Wire-based coronary physiology pullback performed before percutaneous coronary intervention (PCI) discriminates coronary artery disease (CAD) distribution and extent, and is able to predict functional PCI result. No research investigated if quantitative flow ratio (QFR)–based physiology assessment is able to provide similar information.

**Methods:**

In 111 patients (120 vessels) treated with PCI, QFR was measured both before and after PCI. Pre-PCI QFR trace was used to discriminate functional patterns of CAD (focal, serial lesions, diffuse disease, combination). Functional CAD patterns were identified analyzing changes in the QFR virtual pullback trace (qualitative method) or after computation of the QFR virtual pullback index (QVP_index_) (quantitative method). QVP_index_ calculation was based on the maximal QFR drop over 20 mm and the length of epicardial coronary segment with QFR most relevant drop. Then, the ability of the different functional patterns of CAD to predict post-PCI QFR value was tested.

**Results:**

By qualitative method, 51 (43%), 20 (17%), 15 (12%), and 34 (28%) vessels were classified as focal, serial focal lesions, diffuse disease, and combination, respectively. QVP_index_ values >0.71 and ≤0.51 predicted focal and diffuse patterns, respectively. Suboptimal PCI result (post-PCI QFR value ≤0.89) was present in 22 (18%) vessels. Its occurrence differed across functional patterns of CAD (focal 8% vs. serial lesions 15% vs. diffuse disease 33% vs. combination 29%, *p*=0.03). Similarly, QVP_index_ was correlated with post-PCI QFR value (*r*=0.62, 95% CI 0.50–0.72).

**Conclusion:**

Our results suggest that functional patterns of CAD based on pre-PCI QFR trace can predict the functional outcome after PCI.

**Clinical Trial Registration:**

ClinicalTrials.gov, number NCT02811796. Date of registration: June 23, 2016.

**Supplementary Information:**

The online version contains supplementary material available at 10.1007/s10557-021-07162-6.

## Introduction

In the HAWKEYE (Angio-Based Fractional Flow Reserve to Predict Adverse Events After Stent Implantation) study, 16% of investigated vessels had a post-percutaneous coronary intervention (PCI) quantitative flow ratio (QFR) ≤0.89, which was associated with adverse outcome [1]. Algorithms derived from fractional flow reserve (FFR) pullback performed before PCI have been shown to discriminate the pattern of coronary artery disease (CAD) and to predict the post-PCI functional result [2, 3]. Whether an angio-based FFR as QFR is able to provide the same information is unknown.

We therefore performed a proof-of-concept analysis of the HAWKEYE study [1] to assess whether functional patterns of CAD derived from qualitative and quantitative measurements of pre-PCI QFR trace are able to predict post-PCI functional result (QFR≤0.89), which has been associated with outcome in our previous analysis [1].

## Methods

### Study Design

**Fig. 1:**
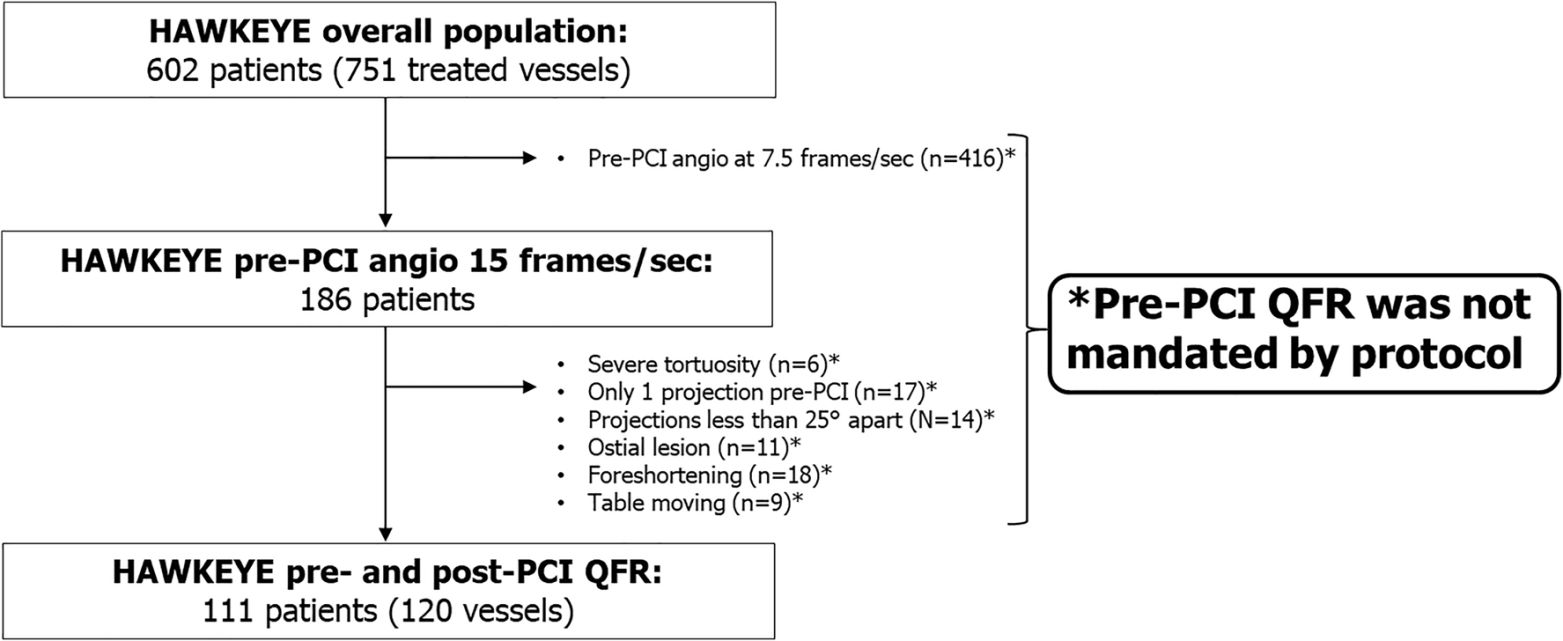
Study flow chart. PCI, percutaneous coronary intervention; sec, seconds; QFR, quantitative flow ratio

This is a post hoc analysis of the HAWKEYE study [1], which prospectively investigated the ability of post-PCI QFR value to discriminate adverse events. When the operator considered the result of the PCI optimized by visual estimation, angiographic images were acquired and sent to the core lab for post-PCI QFR computation [1]. In the main analysis, we analyzed the relationship between post-PCI QFR and outcome [1]. In the present analysis, we retrospectively performed in the central core lab the pre-PCI QFR analysis in all the vessels where projections allowed it. Of note, it is important to clarify that the protocol did not mandate the acquisition of QFR projections pre-PCI and that therefore patients eligible for this substudy are only those whose pre-PCI projections suitable for QFR computation were available (Fig. 1). For this reason, the number of patients included in the present analysis is substantially lower than in the HAWKEYE trial, mainly because most of the participating centers utilized 7.5 frames/second (fps) as default angiography setting rather than 15 fps required for QFR (Fig. 1).

### Study Procedure

All cases were reviewed by two trained members of the core lab and required agreement of both reviewers to be selected for inclusion in the present study. Then, pre-PCI QFR computation and the identification of the functional pattern of CAD by the QFR virtual pullback were performed. Finally, the relationship between functional pattern of CAD and PCI outcome (as assessed by post-PCI QFR) was assessed.

### Quantitative Coronary Analysis and Quantitative Flow Ratio

Quantitative coronary analysis (QCA) and QFR were performed offline, using the software package QAngio XA 3D (Medis Medical Imaging System, Leiden, the Netherlands) in agreement with the step-by-step procedures validated in previous studies [1, 4, 5]. In the present analysis, contrast QFR values were computed both pre- and post-PCI. The QFR value was calculated in the entire vessel from its origin to a distal segment (vessel diameter 1.5 mm) [1, 4]. QFR computation was performed in the core laboratory of the University Hospital of Ferrara by two independent operators certified for QFR computation.

### Definition of Functional Pattern of CAD

Identification of the functional pattern of CAD was performed by the analysis of the trend of pre-PCI QFR virtual pullback (qualitative method) and by calculation of the QFR virtual pullback index (QVP_index_) (quantitative method).

#### Qualitative Method

The reviewers analyzed the presence of step-ups or progressive decline in the QFR virtual pullback. Functional patterns of CAD were defined as follows: (i) focal (presence of single drop ≥0.05 in 10 mm); (ii) serial lesions (presence of 2 or more separated focal drops in the same coronary vessel); (iii) diffuse disease (progressive decline of the QFR value without a clear evidence of focal drop); (iv) combination of previous patterns. Divergences between the reviewers were solved after consensus and with the involvement of a third supervisor.

#### Quantitative Method

The quantitative method was based on the PPG_index_ validated by Collet and colleagues [2]. PPG_index_ was obtained using a motorized FFR pullback to achieve a quantitative classification of the functional pattern of CAD. Similarly, we calculated the QVP_index_ starting from the pre-PCI QFR trace, which automatically generates a point-by-point functional reconstruction of the vessel that is visualized in the trace. The QFR trace is then comparable to an FFR motorized pullback trace. The aim was to objectively describe the functional pattern of CAD. As for the PPG_index_ [2], the QVP_index_ combined the maximal drop of the QFR value over 20 mm (MaxQFR_20mm_) and the length of epicardial coronary segments with QFR deterioration. Specifically, the QVP_index_ was calculated as follows:

$$ \left\{\left({\mathrm{MaxQFR}}_{20\mathrm{mm}}/{\Delta \mathrm{QFR}}_{\mathrm{vessel}}\right)+\right[1-\left[\mathrm{length}\ \mathrm{with}\ \mathrm{functional}\ \mathrm{disease}\ \left(\mathrm{mm}\right)/\mathrm{total}\ \mathrm{vessel}\ \mathrm{length}\ \left(\mathrm{mm}\right)\right]\Big\}/2 $$

MaxQFR_20mm_ was defined as the maximum drop over 20 mm, and delta QFR (ΔQFR) vessel as the difference between QFR values obtained at the ostium of the vessel and at the most distal anatomical location. To obtain these values, the function “index QFR” was used. As previously reported [1, 4], the index QFR is the value of QFR in a specific single point of the vessel and it is easily available moving the relative cursor on the QFR analysis of the vessel. Therefore, modifying the position of the index QFR cursor along the vessel, we were able to obtain every QFR value necessary to the computation. The total vessel length was defined as the distance from the proximal to the distal landmark of the QFR analysis. The length of functional disease was derived selecting the portion of vessel with a decline in the QFR value. As the original PPG_index_ [2], the QVP_index_ is a continuous measure. Values of QVP_index_ approaching 1 were representative of a focal functional pattern of CAD, whereas those near to 0 were suggestive for diffuse functional pattern of CAD.

### Data Collection and Endpoints

Patient demographic data, cardiovascular risk factors, clinical diagnoses, and procedural details were recorded at the time of the PCI. Source data were collected online using dedicated electronic case report forms. Study angiograms were anonymized and submitted to the core laboratory of the University Hospital of Ferrara.

### Statistical Analysis

Continuous data were tested for normal distribution with the Kolmogorov-Smirnov test. Normally distributed values were presented as mean ± SD; otherwise, median value and interquartile range (IQR) were used. Categorical variables were summarized in terms of counts and percentages. Analysis of variance and the Fisher exact test were used to compare quantitative and categorical variables between groups, respectively. For the analyses, vessels were considered independent observations. The Pearson correlations coefficient was used to assess correlation between QVP_index_ and percentage diameter stenosis (%DS) and post-PCI QFR value. Agreement between reviewers in the identification of a functional pattern of CAD was assessed using Cohen’s kappa. The optimal cutoff value of QVP_index_ for the prediction of a different (focal or diffuse) functional pattern of CAD and suboptimal PCI result (post-PCI QFR value ≤0.89) was calculated based on maximizing the sum of sensitivity and specificity, using receiver operating characteristic (ROC) curve analysis. The predictive value of the qualitative functional pattern of CAD or, alternatively, of the QVP_index_ on post-PCI QFR was determined by deriving the standardized β-coefficients in a generalized linear mixed-effects multiple variable regression. To take into account the non-independence of lesions, patient identification was introduced in the multilevel model as random effect, and the model was fitted with random intercepts, as already performed in the main paper of the HAWKEYE study [1]. Bonferroni correction for multiple testing was applied as appropriate. One- or two-tail tests were employed as appropriate, and the statistical significance was defined as *p*<0.05. All analyses were performed with R version 3.5.1 by an independent statistician (MM).

## Results

One hundred eighty-six patients had pre-PCI angiography taken at 15 fps required for QFR. Of these patients, other QFR requirements were not met in 75 patients leaving 111 patients with 120 vessels as the study group (Fig. 1). Patient and vessel characteristics are reported in Table 1. Median pre-PCI QFR was 0.74 [0.70–0.77] and its distribution is shown in Online Figure 1.

**Table 1:** Baseline characteristics

	**Patients (*n*=111)**
Age, years	70 [60–80]
Female sex, no. (%)	36 (32)
BMI, kg/m^2^	26.9 [24.2–30.6]
CV risk factors, no. (%)	
Diabetes	28 (25)
Hypertension	83 (75)
Hyperlipidemia	55 (50)
Current smoker	18 (16)
Medical history, no. (%)	
MI	20 (18)
PCI	19 (17)
CVA	2 (2)
PAD	8 (7)
Chronic kidney disease*	11 (10)
Clinical presentation, no. (%)	
NSTEACS	46 (41)
CCS	65 (59)
Angiographic disease severity	
Multivessel disease, no. (%)	18 (16)
SYNTAX score, point	14 [9–20]
	**Vessels (*n*=120)**
Location, no. (%)	
LAD	70 (58)
LCx	24 (20)
RCA	26 (22)
Pre-PCI QCA and QFR analyses	
RVD, mm	2.8 [2.5–3.4]
Diameter stenosis, %	60 [55–75]
Lesion length, mm	20 [13–25]
QFR, units	0.74 [0.70–0.77]
Procedural data	
Number of stents, no.	1 [1–2]
Diameter of stents, mm	3 [3–3.5]
Total length of stents, mm	25 [18–34]
Post-dilatation, no. (%)	103 (86)
Post-PCI QCA and QFR analyses	
RVD, mm	2.8 [2.5–3.4]
Diameter stenosis, %	10 [8–18]
QFR, units	0.95 [0.91–0.99]
QFR value ≤0.89, no. (%)	22 (18)

*Defined as creatinine ≥2 mg/dl

*BMI* body mass index, *CV* cardiovascular, *MI* myocardial infarction, *PCI* percutaneous coronary intervention, *CVA* cerebrovascular accident, *PAD* peripheral artery disease, *NSTEACS* non-ST-segment elevation acute coronary syndrome, *CCS* chronic coronary syndrome, *SYNTAX* Synergy Between PCI With Taxus and Cardiac Surgery, *LAD* left anterior descending, *LCx* left circumflex, *RCA* right coronary artery, *QCA* quantitative coronary analysis, *QFR* quantitative flow ratio, *RVD* reference vessel diameter

### Description of the Functional Pattern of CAD by Qualitative Method

By qualitative analysis of pre-PCI QFR virtual pullback, 51 (43%) vessels were classified as having a single focal lesion only, 20 (17%) as serial lesions, 15 (12%) as diffuse disease only, and 34 (28%) as a combination of the previous functional patterns of CAD (Visual Overview). The interobserver agreement was excellent (Cohen’s kappa 0.88, 95% CI 0.81–0.95, Online Table 1). Disagreement occurred in one (2%) case of focal pattern, one case (5%) of serial lesions, and 3 (20%) cases of diffuse disease, which were classified as combination of the other functional patterns by the other reviewers (Online Table 1). Anatomical and functional characteristics stratified according to functional patterns of CAD as determined by the qualitative method are shown in Table 2. Both lesion length and pre-PCI QFR values differed across functional patterns of CAD (Table 2 and Online Figure 2). Similarly, the vessel length with functional disease significantly differed between groups (*p*<0.001), being lower in focal and serial lesion patterns (Table 2).

**Table 2:** Anatomical and functional characteristics stratified by functional pattern of CAD

	Focal (*n*=51)	Serial lesions (*n*=20)	Diffuse disease (*n*=15)	Combination (*n*=34)	*p* value
Location, no. (%)					
LAD	26 (51)	15 (75)	7 (47)	22 (64)	
LCx	12 (24)	3 (15)	3 (20)	6 (18)	0.5
RCA	13 (25)	2 (10)	5 (33)	6 (18)	
Pre-PCI quantitative coronary analysis					
RVD, mm	2.8 [2.5–3.3]	2.8 [2.3–3.2]	3 [2.4–3.5]	2.9 [2.5–3.4]	0.9
Diameter stenosis, %	60 [52–74]	63 [52–88]	62 [53–66]	57 [52–63]	0.6
Lesion length, mm	14 [9–18]	22 [20–26]	30 [28–37]	19 [15–25]	<0.001
Pre-PCI quantitative flow ratio					
QFR, units	0.76 [0.73–0.77]	0.71 [0.66–0.76]	0.75 [0.73–0.76]	0.72 [0.70–0.75]	0.004
ΔQFR vessel, units	0.25±0.05	0.28±0.05	0.23±0.08	0.27±0.05	0.01
Total vessel length, mm	65 [60–72]	65 [60–75]	67 [62–70]	68 [61–75]	0.8
Length with functional disease, mm	10 [5–15]	25 [25–30]	45 [30–55]	40 [33–60]	<0.001
QVP_index_, units	0.80±0.09	0.61±0.11	0.38±0.08	0.50±0.10	<0.001
Post-PCI quantitative coronary angiography					
Diameter stenosis, %	10 [5–18]	10 [8–20]	12 [8–20]	10 [7–16]	0.9
Total stented length, mm	19 [16–23]	30 [23–37]	33 [28–40]	29 [23–35]	<0.001
Post-PCI quantitative flow ratio					
QFR, units	0.98 [0.95–1]	0.94 [0.91–0.96]	0.92 [0.89–0.94]	0.93 [0.87–0.96]	<0.001
QFR value ≤0.89, no. (%)	4 (8)	3 (15)	5 (33)	10 (29)	0.03

*LAD* left anterior descending, *LCx* left circumflex, *RCA* right coronary artery, *PCI* percutaneous coronary intervention, *RVD* reference vessel diameter, *QFR* quantitative flow ratio, *ΔQFR* delta QFR vessel, *QVP*_*index*_ QFR virtual pullback index

### Description of the Functional Pattern of CAD by Quantitative Method

Mean QVP_index_ was 0.63±0.18 (range 0.27–0.97) and its distribution is shown in Online Figure 3, while QVP_index_ values across different functional patterns of CAD are shown in Online Figure 4. QVP_index_ did not correlate with the pre-PCI %DS, as assessed by QCA. ROC curve analyses of QVP_index_ for the prediction of focal and diffuse pattern of CAD are shown in Online Figures 5 and 6.

### Functional Pattern of CAD and Post-PCI QFR Value

**Fig. 2:**
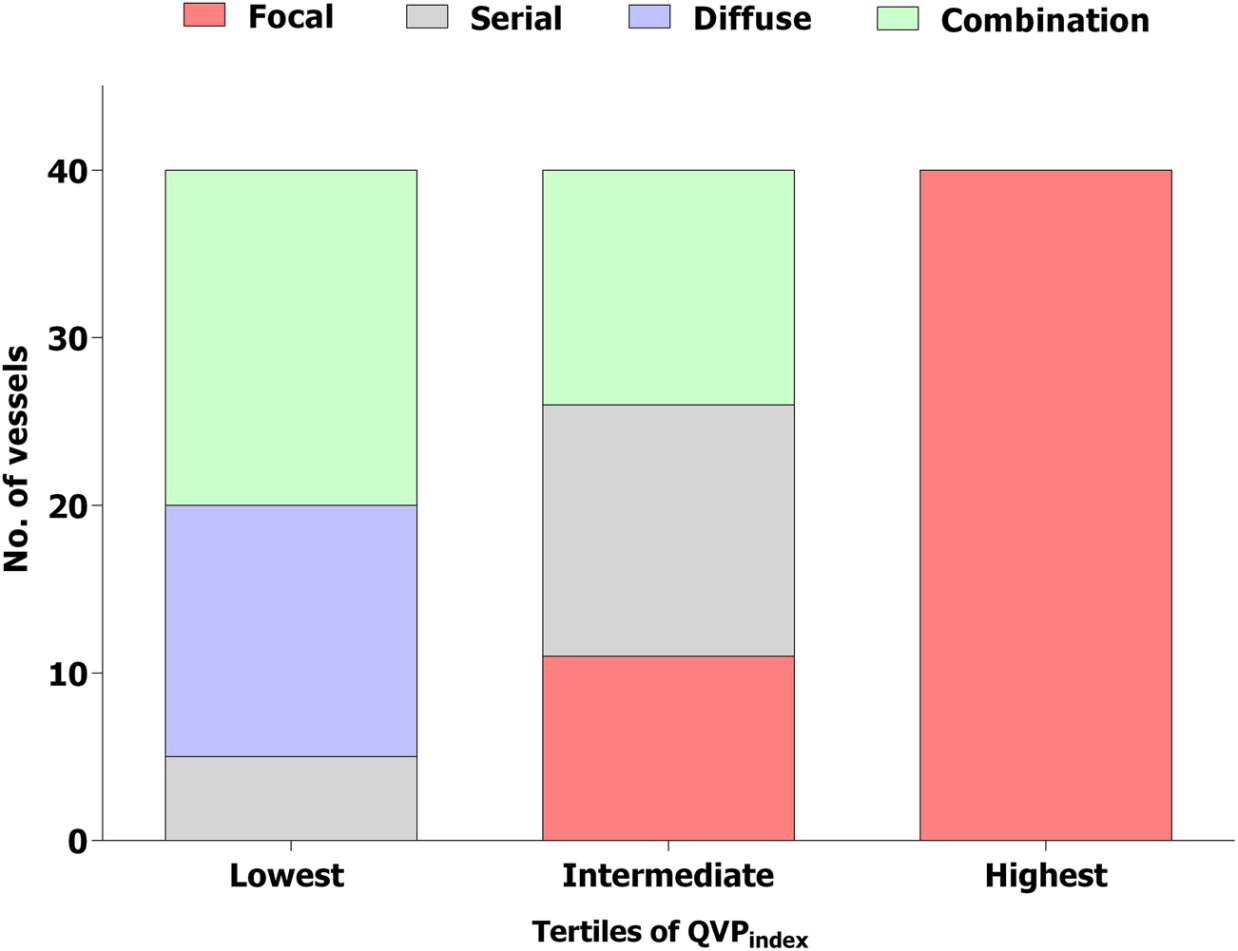
Distribution of functional patterns of CAD in tertiles of QVP_index_. The colors indicate the functional patterns of CAD (as assessed by qualitative method) in QVP_index_ tertiles (lowest tertiles ≤0.54, intermediate tertile 0.55–0.71, highest tertile >0.71). No., number; CAD, coronary artery disease; QFR, quantitative flow ratio; QVP_index_, QFR virtual pullback index

**Fig. 3:**
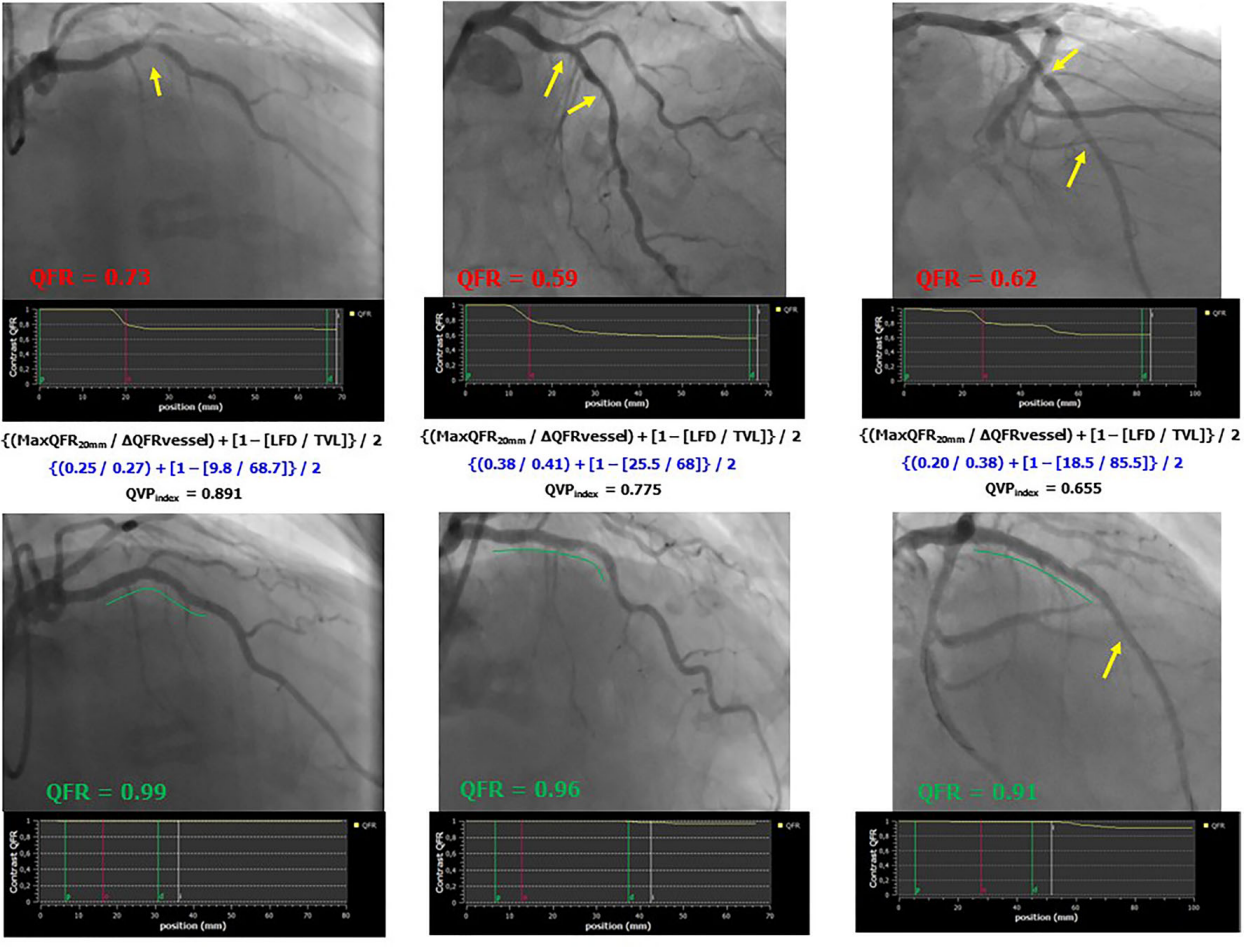
Examples of successful PCI in vessels with intermediate-high QVP_index_. QFR, quantitative flow ratio; MaxQFR_20mm_, maximal drop of QFR value in 200 mm; ΔQFRvessel, difference in the QFR values from the beginning to the end of the vessel; LFD, length of the vessel with functional disease; TVL, total vessel length; QVP_index_, QFR virtual pullback index; PCI, percutaneous coronary intervention. Yellow arrows indicate the presence of coronary lesions before or after PCI, whereas green lines indicate stented segments in post-PCI images

**Fig. 4:**
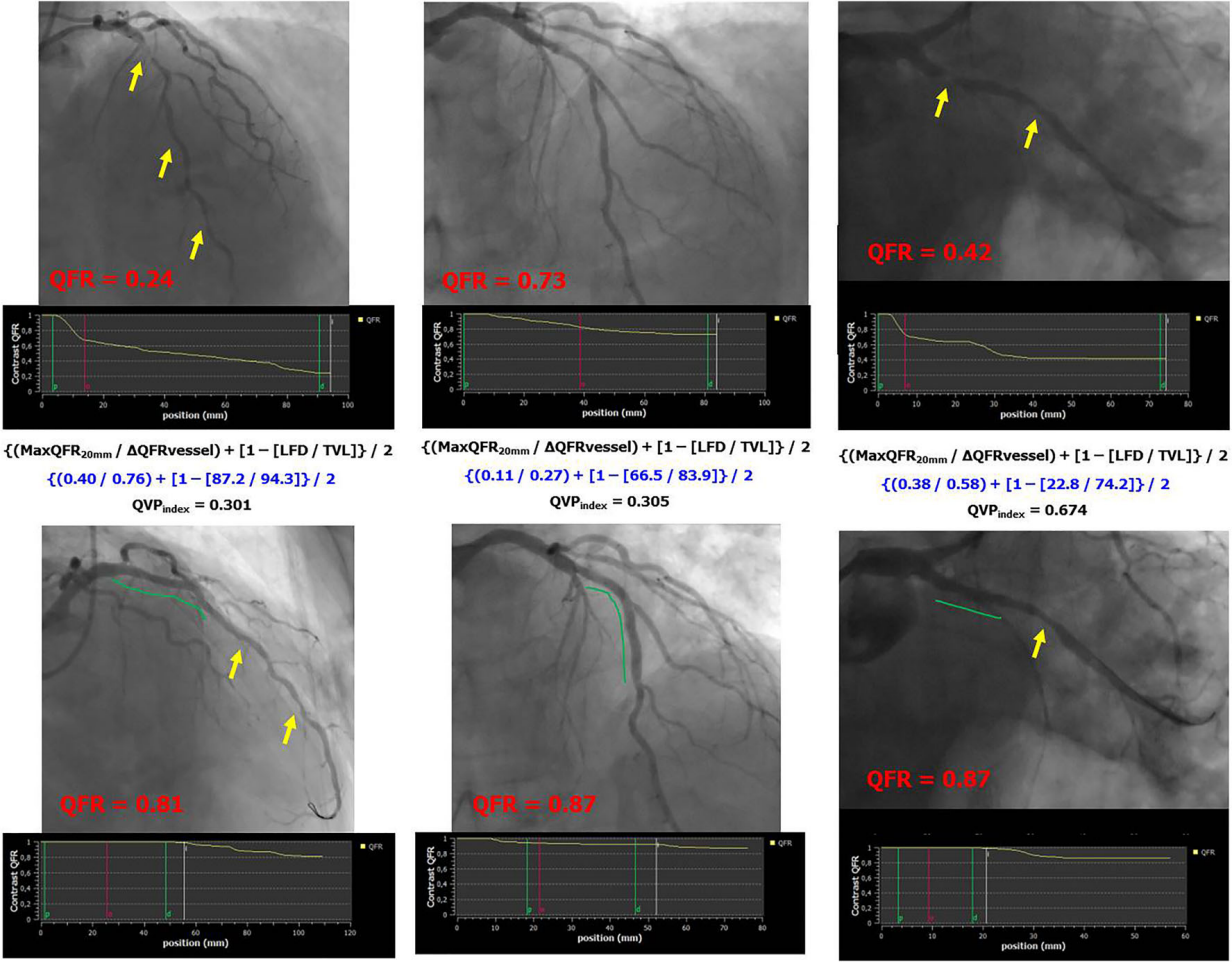
Examples of suboptimal PCI in vessels with intermediate-low QVP_index_. QFR, quantitative flow ratio; MaxQFR_20mm_, maximal drop of QFR value in 200 mm; ΔQFRvessel, difference in the QFR values from the beginning to the end of the vessel; LFD, length of the vessel with functional disease; TVL, total vessel length; QVP_index_, QFR virtual pullback index; PCI, percutaneous coronary intervention. Yellow arrows indicate the presence of coronary lesions before or after PCI, whereas green lines indicate stented segments in post-PCI images

The median post-PCI QFR value was 0.95 [0.91–0.99]. PCI procedure was suboptimal (post-PCI QFR ≤0.89) in 22 (18%) vessels. Post-PCI QFR values significantly differed across the functional patterns of CAD (qualitative method) (Table 2 and Online Figure 7). After correction for potential confounding factors, focal pattern was associated with higher post-PCI QFR value (Std *β* 0.802, 95% CI 0.487 to 1.118, *p*<0.001), whereas diffuse pattern was associated with lower post-PCI QFR value (Std *β* −0.467, 95% CI −0.892 to −0.042, *p*=0.03). The occurrence of suboptimal PCI differed between functional patterns of CAD (Table 2). Vessels with focal or serial lesion patterns showed a lower occurrence of suboptimal PCI (8% and 15%, respectively), whereas it was higher in the presence of diffuse disease or combination patterns (29% and 33%, respectively) (Table 2, *p*=0.03). QVP_index_ correlated with post-PCI QFR value (*r*=0.62, 95% CI 0.50–0.72). Multiple regression analysis confirmed that higher QVP_index_ values were significant predictors of higher post-PCI QFR values (Std 0.713, 95% CI 0.552 to 0.873, *p*<0.001). According to QVP_index_ tertiles, post-PCI QFR was 0.91 [0.85–0.93] in the lowest, 0.94 [0.91–0.97] in the intermediate, and 0.99 [0.97–1] in the highest (*p*<0.001) (Fig. 2 and Online Figure 8). PCI result was suboptimal in 15 (37%), 5 (12%), and 2 (5%) vessels of lowest, intermediate, and highest QVP_index_ tertiles, respectively (*p*<0.001). A QVP_index_ ≤0.57 predicted suboptimal PCI outcome with sensitivity of 77% and specificity of 72% (C-statistics 0.80, 95% CI 0.71–0.86, Online Figure 9). Examples of different functional patterns of CAD with the corresponding QVP_index_ and PCI outcome are reported in Figs. 3 and 4.

## Discussion

The present post hoc analysis of the HAWKEYE study showed that the functional pattern of CAD evaluated both qualitatively and quantitatively on the basis of pre-PCI QFR trace has a strong correlation with post-PCI QFR value and can predict post-PCI suboptimal functional outcome.

Since the first years of interventional cardiology, several attempts to discriminate the distribution of coronary atherosclerosis have been made. The primary aim was to classify the pattern of CAD to discriminate the preferred treatment strategy, and estimate the risk of PCI complications and long-term prognosis. Intracoronary physiology has been introduced into daily practice to discriminate between vessels that would benefit from revascularization vs. those with diffuse disease where percutaneous intervention may not be useful. FFR pullback has been shown to be feasible and reproducible in the discrimination of the CAD pattern [2, 3]. Similar evidence has been provided using non-hyperemic pressure ratios (NHPRs) [6, 7]. However, invasive physiology tools have displayed some limitations [8] including time needed for the procedure including wiring and pullback, and, in the case of FFR, the intravenous infusion of adenosine. QFR, on the other hand, does not require wiring or drug infusion and, after adequate training, the analysis can be performed in around 5 min [1, 4]. Another interesting avenue is represented by the development of a computed tomography–derived FFR-based interactive planner which has the potential to improve non-invasively diagnostic and therapeutic interventions [9, 10].

The present post hoc analysis of the HAWKEYE trial utilized pre-PCI QFR virtual pullback for the identification of the functional pattern of CAD. The discrimination was performed both by the analysis of drops and/or progressive decline in the pre-PCI QFR virtual pullback and by a quantitative method, namely the QVP_index_.

The major clinical implication of the identification of the functional pattern of CAD is related to the possibility to plan PCI in advance or to avoid it when an optimal functional result is unlikely. In the HAWKEYE trial, we found that post-PCI QFR values ≤0.89 were associated with an increased risk of vessel-related cardiac events [1]. Lower post-PCI values were due to either focal instent drop or untreated lesions or presence of residual diffuse disease [1]. The present post hoc analysis shows that these suboptimal results could have been anticipated by pre-PCI QFR analysis.

The focal pattern of CAD, evaluated both qualitatively and quantitatively, showed the highest probability to achieve an optimal result with PCI and stent implantation. In contrast, suboptimal PCI was more frequent in vessels with diffuse disease or a combination of patterns. The amount of vessel length with diffuse disease, which is well described by lowest QVP_index_, was strongly associated with suboptimal PCI functional outcome. Stent implantation within a portion of the most highly diseased diffuse area tends to increase residual QFR value, but frequently it is not enough to achieve good functional results. If surgical revascularization or different secondary prevention strategies may be effective or preferable for these patients is unclear and future trials are warranted. In daily practice, we may speculate that a careful analysis of the pre-PCI QFR virtual pullback will help operators in the development of the proper revascularization strategy and in the optimization of PCI results.

### Limitations

The present investigation has several limitations that should be considered. First, pre-PCI QFR was not mandated by protocol. Therefore, it was available in a relatively low percentage of patients from the HAWKEYE study. The primary reason for inability to perform QFR pre-PCI was that most of the participating centers utilized 7.5 fps as default angio setting with 15 fps required to perform QFR. Further other QFR requirements such as 2 angiographic projections greater than 25° apart were not observed. It should be emphasized that the relatively smaller study population should not be considered as a technical failure to obtain QFR but rather to the fact that pre-PCI QFR was not mandated by the study, and angiographic views and use of 15 fps were not performed pre-PCI routinely based on the study protocol (Fig. 1) [1]. Therefore, our findings should be considered hypothesis-generating and should be replicated in dedicated prespecified subsets. Second, QFR computation was performed offline by experienced and trained operators. Although previous studies have shown a good agreement between offline and online measurements, further confirmations are warranted [5]. Similarly, if QVP_index_ computation can be automatized should be properly investigated. Third, a complete validation of the QVP_index_ requires a head-to-head comparison with the original PPG_index_ derived by motorized FFR pullback. However, the present analysis should be considered proof-of-concept of an alternative, quick, easy tool for the assessment of the functional pattern of CAD and further studies are necessary to validate its application in daily practice. Finally, the idea of “QFR-guided virtual PCI” should be considered a hypothesis and prospective appropriate studies are needed to validate its feasibility and effectiveness in daily practice.

## Conclusions

The functional pattern of CAD evaluated both qualitatively and quantitatively utilizing pre-PCI QFR trace is able to predict post-PCI functional results. In vessels with focal and serial lesion functional patterns of CAD, PCI can achieve optimal results. On the contrary, vessels with diffuse disease are more challenging and future studies are needed to understand how to optimize their management and prognosis.

Visual overview. Distribution of functional pattern of CAD. The central pie chart represents the functional patterns of CAD as assessed by the analysis of the drops and the decline of pre-PCI QFR virtual pullback (qualitative method). CAD, coronary artery disease; QFR, quantitative flow ratio

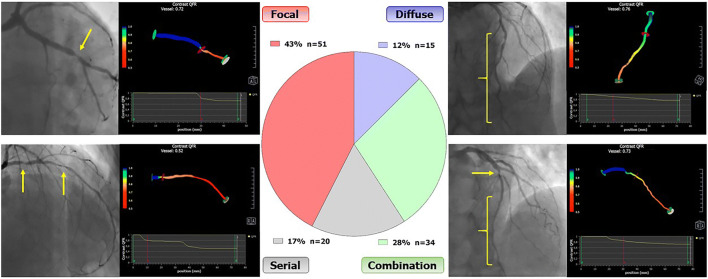



## Supplementary Information

ESM 1 (DOC 1660 kb)

## Data Availability

Data will be available upon reasonable request.

## Abbreviations

QFR: Quantitative flow ratio
HAWKEYE: Angio-Based Fractional Flow Reserve to Predict Adverse Events After Stent Implantation
FFR: Fractional flow reserve
iFR: Instantaneous wave-free ratio
CAD: Coronary artery disease
PCI: Percutaneous coronary intervention
QVP_index_: QFR virtual pullback index

## Code Availability

R version 3.5.1.
