# Supplementary material for: Angio-Based Fractional Flow Reserve, Functional Pattern of Coronary Artery Disease, and Prediction of Percutaneous Coronary Intervention Result: a Proof-of-Concept Study
Source: Cardiovasc Drugs Ther. 2021 Apr 8;36(4):645–53. doi: 10.1007/s10557-021-07162-6 (PMC9270302; doi:10.1007/s10557-021-07162-6)
Supplement: Supplementary file 1 — (DOC 1660 kb) [file 10557_2021_7162_MOESM1_ESM.doc]

**Supplemental online**

**Angio-based Fractional Flow Reserve, Functional Pattern of Coronary Artery Disease, and Prediction of Percutaneous Coronary Intervention Result: a proof-of-concept study.NCT02811796**

**INDEX**

- Page 2: Supplemental Figure 1. Distribution of pre-PCI QFR values
- Page 3: Supplemental Table 1. Inter-observer agreement on functional patterns of CAD based on visual assessment of QFR trace (qualitative method)
- Page 4: Supplemental Figure 2. Pre-PCI QFR values across different functional patterns of CAD
- Page 5: Supplemental Figure 3. Distribution of QVP_index_ values
- Page 6: Supplemental Figure 4. QVP_index_ values across different functional patterns of CAD
- Page 7: Supplemental Figure 5. ROC curve analysis of QVP_index_ for the prediction of the focal functional pattern of CAD
- Page 8: Supplemental Figure 6. ROC curve analysis of QVP_index_ for the prediction of the diffuse disease functional pattern of CAD
- Page 9: Supplemental Figure 7. Post-PCI QFR values across different functional patterns of CAD
- Page 10: Supplemental Figure 8. Post-PCI QFR values across QVP_index_ tertiles
- Page 11: Supplemental Figure 9. ROC curve analysis of QVP_index_ for the prediction of suboptimal PCI outcome (as defined by post-PCI QFR value ≤0.89)

**Supplemental Figure 1. Distribution of pre-PCI QFR values**

**
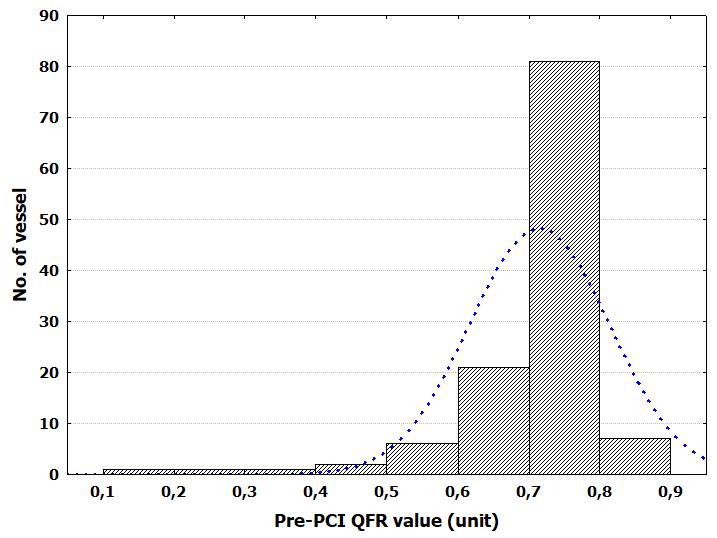
**

No: number. PCI: percutaneous coronary intervention. QFR: quantitative flow ratio.

**Supplemental Table 1. Inter-observer agreement on functional patterns of CAD based on visual assessment of QFR trace (qualitative method)**

|  |  | **Reviewer #1** | | | |
| --- | --- | --- | --- | --- | --- |
|  |  | **Focal** | **Serial lesions** | **Diffuse** | **Combination** |
| **Reviewer #2** | **Focal** | 50 | 0 | 0 | 1 |
|  | **Serial lesions** | 0 | 19 | 0 | 1 |
|  | **Diffuse** | 0 | 0 | 12 | 3 |
|  | **Combination** | 1 | 1 | 3 | 29 |
|  |  |  |  |  |  |

Cohen’s Kappa 0.88 (95%CI 0.81-0.95

Standard error of Kappa 0.036

The calculations above only consider exact matches (n=110, 91.7% of the observations) between observers. This is due to the fact that we may not order categories and we may not consider close matches.

CAD: coronary artery disease. QFR: quantitative flow ratio.

**Supplemental Figure 2. Pre-PCI QFR values across different functional patterns of CAD**

**
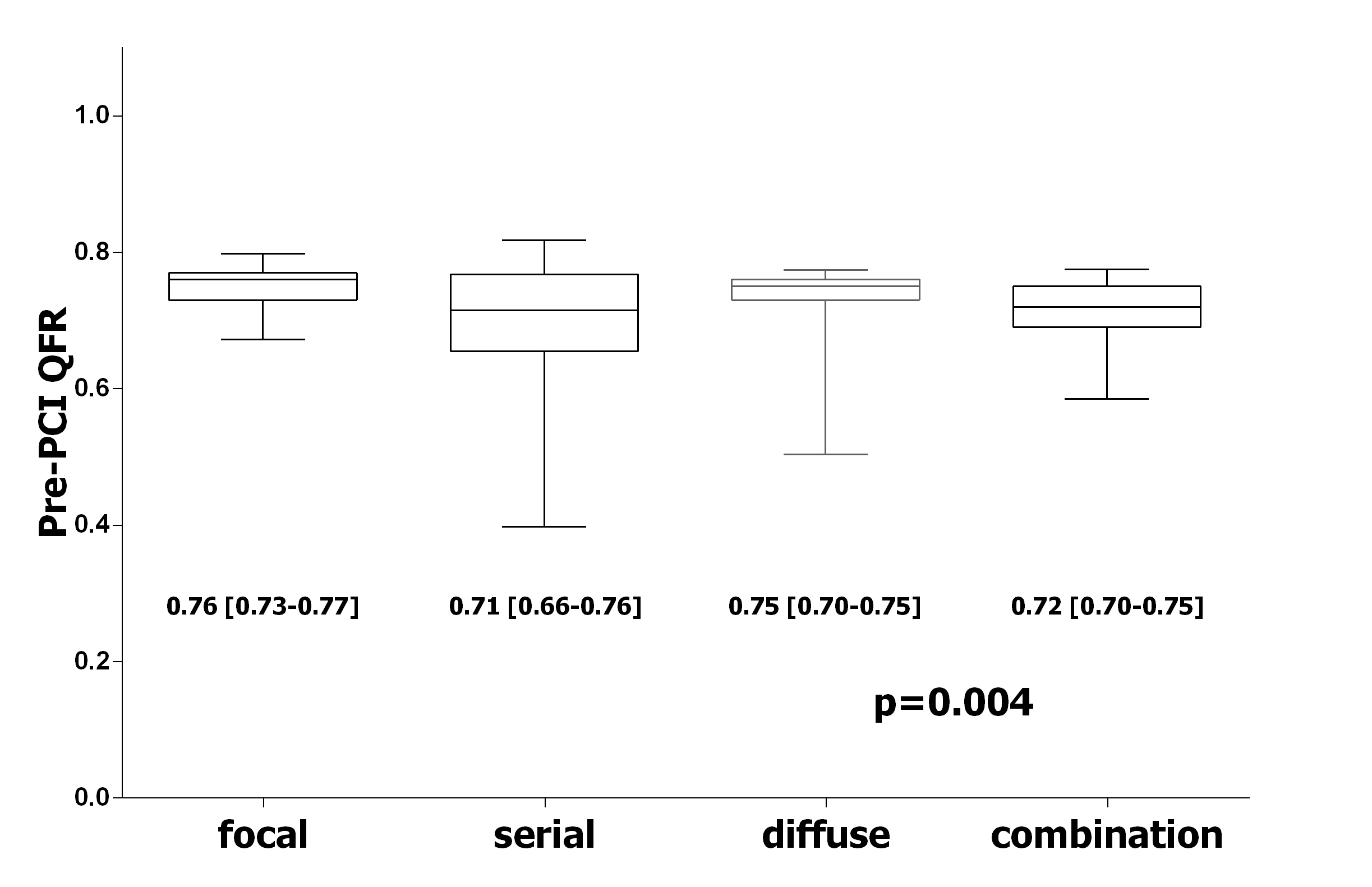
**

PCI: percutaneous coronary intervention. QFR: quantitative flow ratio. CAD: coronary artery disease.

**Supplemental Figure 3. Distribution of QVP_index_ values**


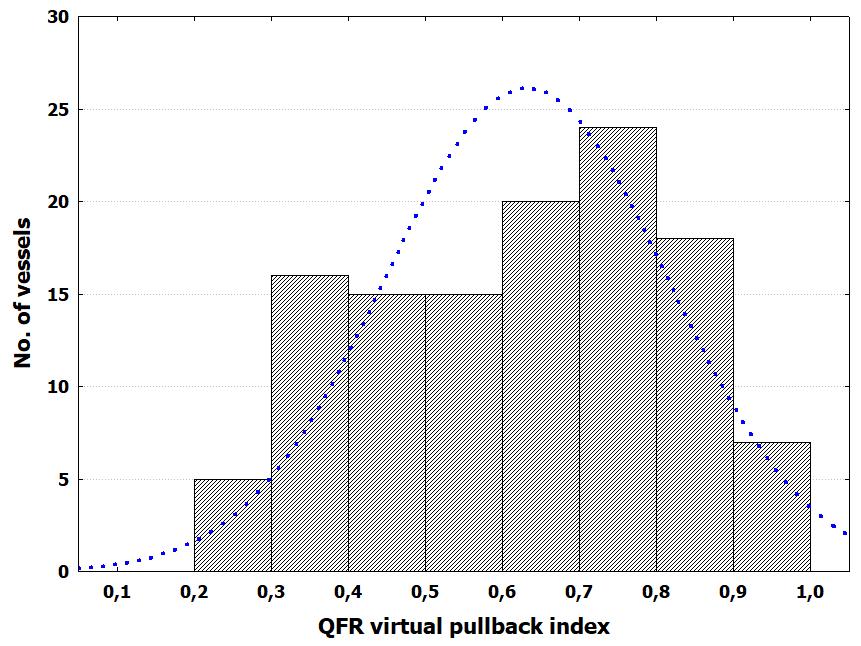


No. number. QFR: quantitative flow ratio.

**Supplemental Figure 4. QVP_index_ values across different functional patterns of CAD**

**
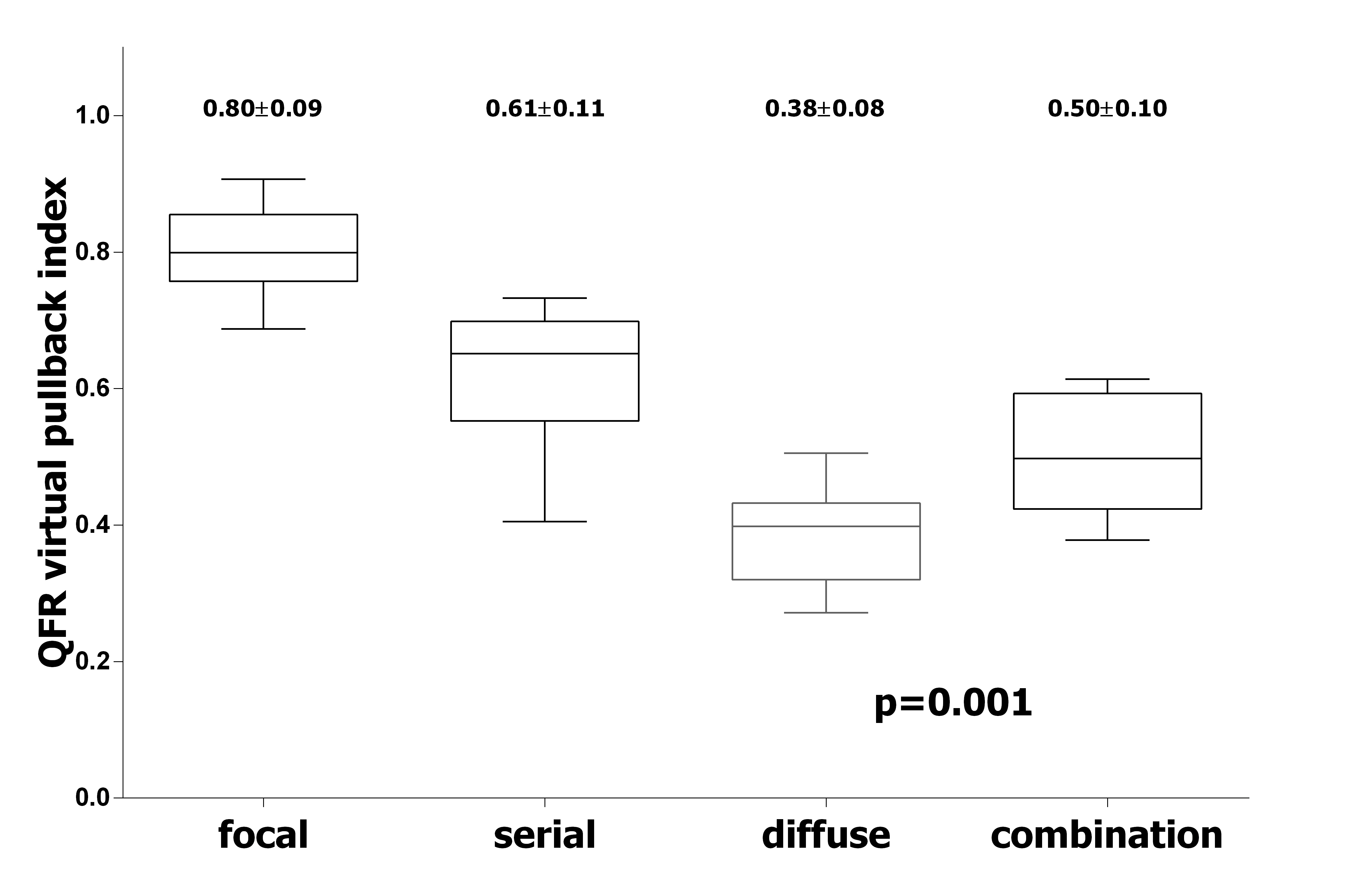
**

QFR: quantitative flow ratio. CAD: coronary artery disease.

**Supplemental Figure 5. ROC curve analysis of QVP_index_ for the prediction of the focal functional pattern of CAD**

Area under the curve: 0.97, 95% confidence interval 0.92 to 0.99

Best cut-off of QVP_index_ for the prediction of the focal pattern >0.71

ROC: receiver-operating characteristics. QFR: quantitative flow ratio virtual pullback. CAD: coronary artery disease.

**Supplemental Figure 6. ROC curve analysis of QVP_index_ for the prediction of the diffuse disease functional pattern of CAD**

Area under the curve: 0.92, 95% confidence interval 0.85 to 0.96

Best cut-off of QVP_index_ for the prediction of the diffuse pattern ≤0.51

ROC: receiver-operating characteristics. QFR: quantitative flow ratio virtual pullback. CAD: coronary artery disease.

**Supplemental Figure 7. Post-PCI QFR values across different functional patterns of CAD**

**
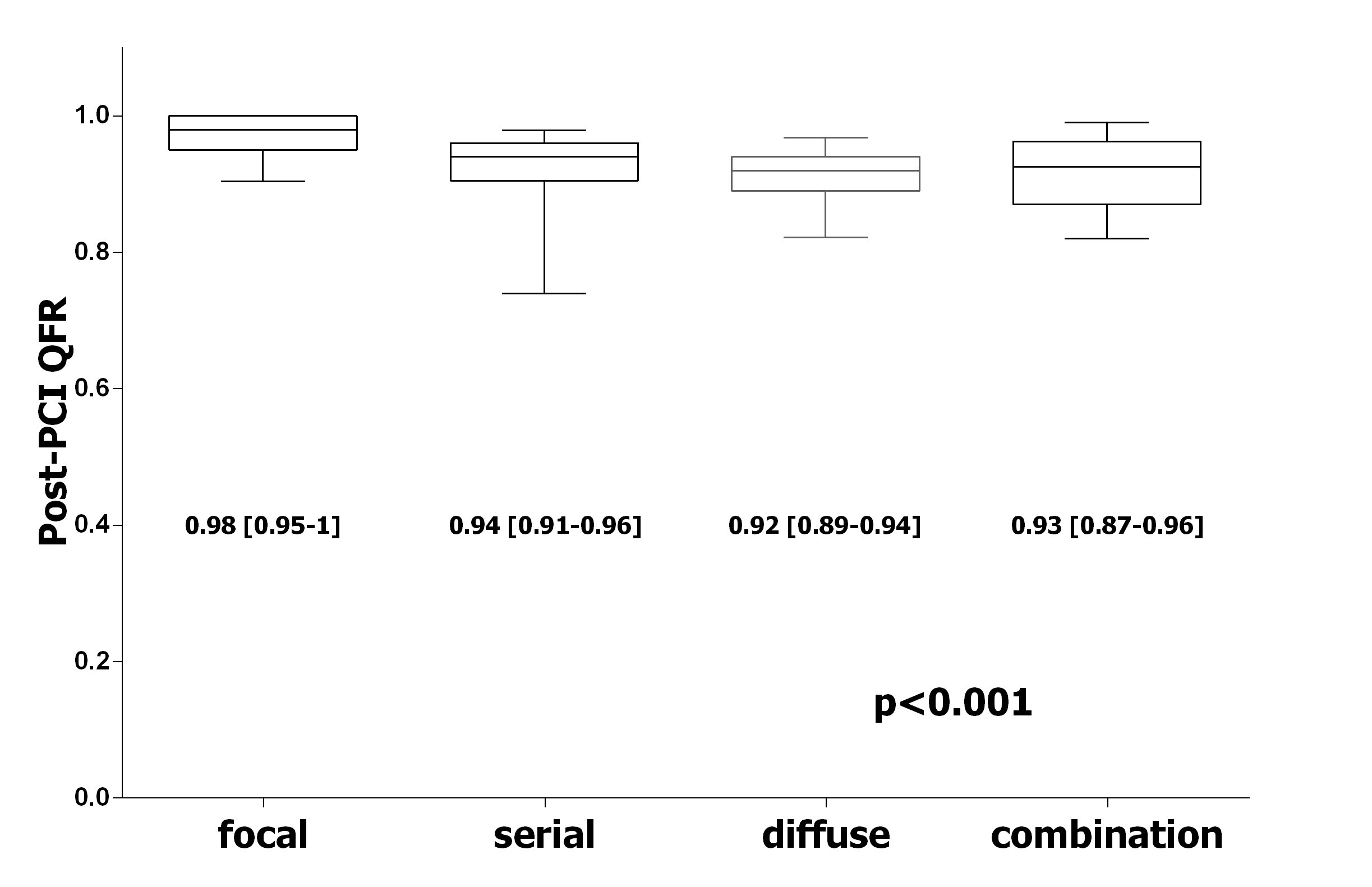
**

PCI: percutaneous coronary intervention. QFR: quantitative flow ratio.

**Supplemental Figure 8. Post-PCI QFR values across QVP_index_ tertiles**

**
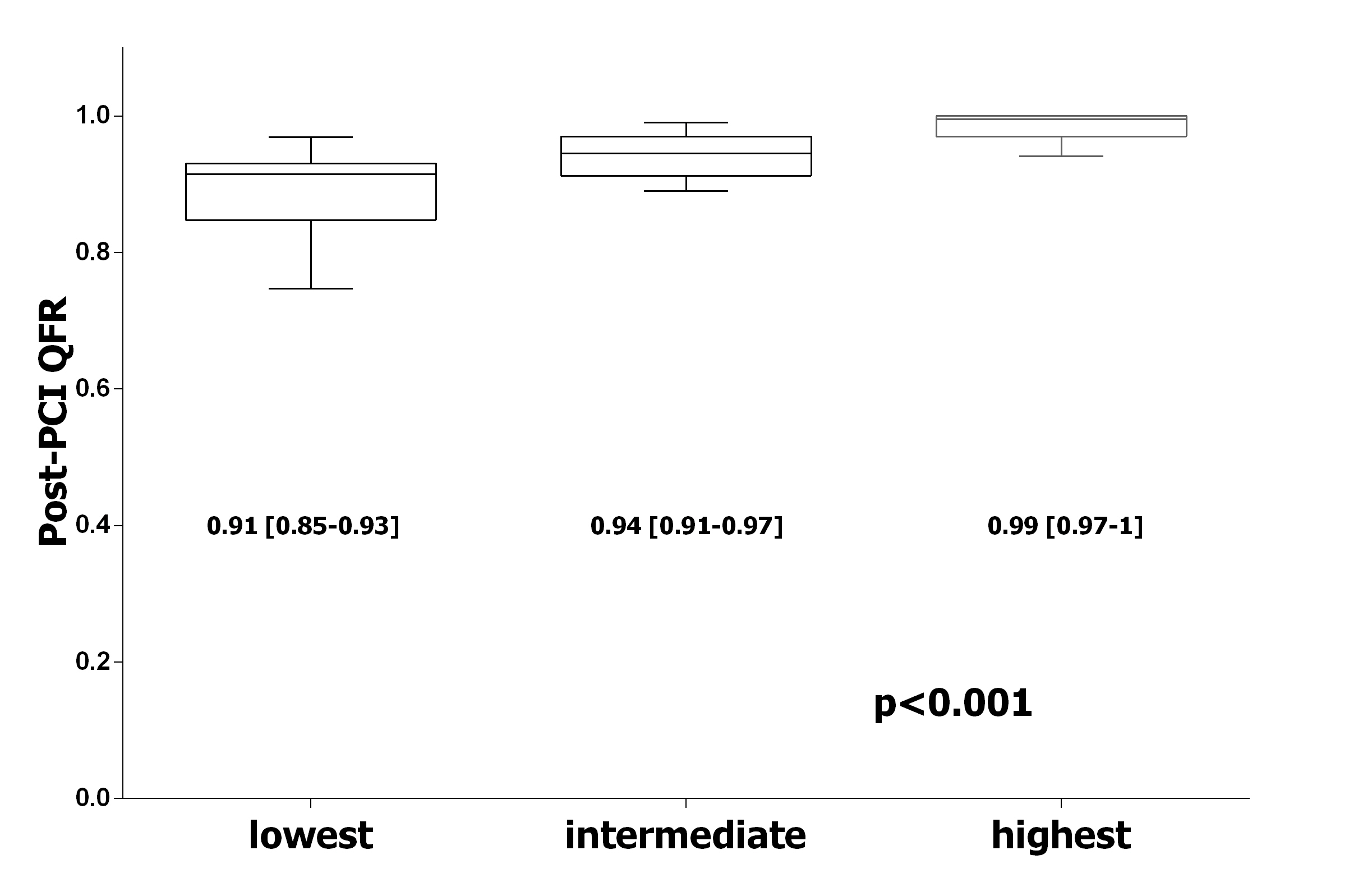
**

PCI: percutaneous coronary intervention. QFR: quantitative flow ratio. QVP: quantitative flow ratio virtual pullback

**Supplemental Figure 9. ROC curve analysis of QVP_index_ for the prediction of suboptimal PCI outcome (as defined by post-PCI QFR value ≤0.89)**

Area under the curve: 0.80, 95% confidence interval 0.71 to 0.86

Best cut-off of QVP_index_ for the prediction of the diffuse pattern ≤0.57

ROC: receiver-operating characteristics. QFR: quantitative flow ratio. QVP: quantitative flow ratio virtual pullback. CAD: coronary artery disease.
